# Biological Control of Lettuce Drop and Host Plant Colonization by Rhizospheric and Endophytic Streptomycetes

**DOI:** 10.3389/fmicb.2016.00714

**Published:** 2016-05-20

**Authors:** Xiaoyulong Chen, Cristina Pizzatti, Maria Bonaldi, Marco Saracchi, Armin Erlacher, Andrea Kunova, Gabriele Berg, Paolo Cortesi

**Affiliations:** ^1^Department of Food, Environmental and Nutritional Sciences, University of MilanMilan, Italy; ^2^Institute of Environmental Biotechnology, Graz University of TechnologyGraz, Austria

**Keywords:** biocontrol, hazard ratio, lettuce, *Sclerotinia sclerotiorum*, *Streptomyces*, rhizosphere competence, endophytes

## Abstract

Lettuce drop, caused by the soil borne pathogen *Sclerotinia sclerotiorum*, is one of the most common and serious diseases of lettuce worldwide. Increased concerns about the side effects of chemical pesticides have resulted in greater interest in developing biocontrol strategies against *S. sclerotiorum*. However, relatively little is known about the mechanisms of *Streptomyces* spp. as biological control agents against *S. sclerotiorum* on lettuce. Two *Streptomyces* isolates, *S. exfoliatus* FT05W and *S. cyaneus* ZEA17I, inhibit mycelial growth of *Sclerotinia sclerotiorum* by more than 75% *in vitro*. We evaluated their biocontrol activity against *S. sclerotiorum in vivo*, and compared them to *Streptomyces lydicus* WYEC 108, isolated from *Actinovate*®. When *Streptomyces* spp. (10^6^ CFU/mL) were applied to *S. sclerotiorum* inoculated substrate in a growth chamber 1 week prior lettuce sowing, they significantly reduced the risk of lettuce drop disease, compared to the inoculated control. Interestingly, under field conditions, *S. exfoliatus* FT05W and *S. cyaneus* ZEA17I protected lettuce from drop by 40 and 10% respectively, whereas *S. lydicus* WYEC 108 did not show any protection. We further labeled *S. exfoliatus* FT05W and *S. cyaneus* ZEA17I with the enhanced GFP (EGFP) marker to investigate their rhizosphere competence and ability to colonize lettuce roots using confocal laser scanning microscopy (CLSM). The abundant colonization of young lettuce seedlings by both strains demonstrated *Streptomyces*' capability to interact with the host from early stages of seed germination and root development. Moreover, the two strains were detected also on 2-week-old roots, indicating their potential of long-term interactions with lettuce. Additionally, scanning electron microscopy (SEM) observations showed EGFP-*S. exfoliatus* FT05W endophytic colonization of lettuce root cortex tissues. Finally, we determined its viability and persistence in the rhizosphere and endorhiza up to 3 weeks by quantifying its concentration in these compartments. Based on these results we conclude that *S. exfoliatus* FT05W has high potential to be exploited in agriculture for managing soil borne diseases barely controlled by available plant protection products.

## Introduction

The world population will continue to grow until at least 2050, and possibly increase from 7 to 11 billion people (Van Den Bergh and Rietveld, 2004). For this reason, food security has become one of the main challenges to human development, and therefore any plant pathogen causing substantial crop yield losses needs to be minimized. Drop, caused by *Sclerotinia* species, is globally one of the most destructive soil borne diseases of important horticultural crops. Three are the possible *Sclerotinia* species involved in lettuce drop, *S. sclerotiorum, S. minor*, and *S. nivalis* (Van Beneden et al., 2009). On lettuce, the pathogens can survive in the soil as sclerotia for years, or as mycelium on dead plants. *Sclerotinia* can infect the lettuce crown, roots, and leaves at any stage of plant development (Rabeendran et al., 2006). The hyphae arising from sclerotia penetrate lettuce directly through senescent leaves and root tissues, and can cause wilting and complete plant collapse in less than 2 days (Subbarao, 1998). In Lombardy, northern Italy, commercial lettuce cultivation is threatened by *S. sclerotiorum* infections (Bonaldi et al., 2014) and different strategies and methods are being used to prevent and manage lettuce drop epidemics. So far, fungicides have been extensively used, however, the adverse side effects of chemicals represent a serious threat to living organisms including human and the environment (Kohler and Triebskorn, 2013; Lamberth et al., 2013). In addition, for many plant pathogens, fungicide resistant populations have made many molecules ineffective. Therefore, there is an increasing demand for alternative and sustainable methods of disease management (Spadaro and Gullino, 2004; Ishii, 2006). An up-and-coming alternative to chemicals is the use of biological control agents (BCAs). *Coniothyrium, Trichodema, Bacillus*, and *Pseudomonas* spp. have been used for the management of numerous diseases (Walsh et al., 2001; Howell, 2003; Jacobsen et al., 2004). In comparison to these well-known BCAs, there is only limited application of *Streptomyces* in agriculture, contrary to its exploitation in pharmaceutical industry.

*Streptomyces* are Gram-positive bacteria ubiquitously found in soil, where they significantly contribute to the turnover of organic matter. They are the largest genus of Streptomycetaceae family (order Actinomycetales), comprising more than 500 species (Labeda et al., 2012). Very few species are pathogenic to human or plants. *S. scabies* and *S. turgidiscabies* cause scab disease on tuber and taproot crops, such as potatoes, sweet potatoes, carrots or beet (Lehtonen et al., 2004; Loria et al., 2006). On the contrary, many species produce a variety of bioactive secondary metabolites and enzymes, which gives them potential in biocontrol and plant growth promotion. It has been hypothesized that high levels of antagonistic *Streptomyces* in naturally-occurring or induced suppressive soils significantly contribute to disease suppression (Kinkel et al., 2012). Similarly, organic soil amendments resulted in shift and increase of the density of indigenous *Streptomyces* populations and led to disease suppression (Cohen et al., 2005; Mazzola and Zhao, 2010). The current research, however, focused mainly on evaluating biocontrol activity of individual antagonistic *Streptomyces* spp.: *S. globisporus* JK-1 inhibited *Pyricularia oryzae*, reducing thus rice blast severity (Li et al., 2011); *S. rochei* ACTA1551 protected tomato seeds from *F. oxysporum* infection (Kanini et al., 2013); the metabolites of *S. bikiniensis* HD-087 effectively suppressed *F. oxysporum* and induced resistance in cucumber (Zhao et al., 2012); three endophytic *Streptomyces* isolates significantly promoted tomato plant growth by producing auxins and siderophores (Verma et al., 2011). Until now, only few commercial *Streptomyces-*based biocontrol products have been developed for the market, e.g., *Mycostop*® based on *S. griseoviridis* strain K61, or *Actinovate*® and *Micro108*® based on *S. lydicus* strain WYEC 108 (Palaniyandi et al., 2013). They showed moderate protection of different plants against various pathogens (Paulitz and Belanger, 2001; Zeng et al., 2012; Tian and Zheng, 2013). Although vast array of secondary metabolites have been assumed to act in the biocontrol and plant growth promoting activity of streptomycetes (Trejo-Estrada et al., 1998; Prapagdee et al., 2008; Schrey and Tarkka, 2008; Tarkka and Hampp, 2008), only in few cases the exact mechanism was elucidated, e.g., disruption of geldanamycin production in recombinant *S. melanosporofaciens* strain FP-60 resulted in the loss of its activity against *S. scabies* (Agbessi et al., 2003), or the involvement of siderophores in rice growth promotion by *Streptomyces* sp. GMKU 3100 (Rungin et al., 2012). Moreover, priming by streptomycetes to activate plant defense responses through induced and/or acquired systemic resistance pathways could be an additional mechanism of action involved in disease suppression (Conn et al., 2008; Lehr et al., 2008; Kurth et al., 2014; Salla et al., 2016).

Plant roots are colonized by vast amount of microbes, some of which contribute to biological control (Whipps, 2001; Hardoim et al., 2015). The complex community of microbes produces a variety of compounds and develops interactions, including the competition between BCAs and plant pathogens (Raaijmakers et al., 2009). The rhizosphere—a layer of the soil surrounding the root surface including rhizoplane—harbors an array of microorganisms, whose composition is influenced by root exudates (Hiltner, 1904; Lugtenberg and Kamilova, 2009). Rhizosphere competence is a prerequisite for a BCA to establish beneficial relationship with the host. In fact, some rhizobacteria successfully colonizing rhizosphere protected the host from soil borne fungal pathogens (Kloepper et al., 2004; Haas and Defago, 2005; Weller, 2007). Nowadays, several genetic markers are available for the identification and quantification of microorganisms in the rhizosphere as well as in the endorhiza—the plant inner root area. Among these, antibiotic resistance has been used as a marker to quantify the colonization dynamics of microbes in the plant root system (Gamalero et al., 2003; Adesina et al., 2009; Angelopoulou et al., 2014; Schreiter et al., 2014; Bonaldi et al., 2015). At the same time, fluorescent proteins, such as the green fluorescent protein (GFP), provide appropriate tool to monitor the colonization patterns of BCAs on plants. Enhanced GFP (EGFP), a modified version of GFP, has numerous silent nucleotide substitutions to maximize its expression in mammalian cells (Haas et al., 1996), and is also suitable for use in *Streptomyces* spp. because of a similar codon usage (Sun et al., 1999). GFP tagging was frequently used to determine colonization of host by beneficial *Bacillus* and *Pseudomonas* species (Krzyzanowska et al., 2012; Li et al., 2013; Subramanian et al., 2015). However, up to now, very few studies have addressed the plant colonization by EGFP-tagged *Streptomyces* spp. The strain EN 27 colonized the inner seed area of wheat at early stage of development (Coombs and Franco, 2003) and the pathogenic strain *S. turgidiscabies* Car8 colonized several-day-old radish seedlings (Joshi et al., 2007). For BCAs, the viability and persistence in rhizosphere and endorhiza are pre-requisites for their application against soil borne pathogens. In fact, certain biocontrol rhizobacteria showed stable and long-term colonization of the root surface, as well as endophytic colonization (Compant et al., 2005; Berg, 2009). Therefore, determining the rhizosphere competence and endophytic colonization of the host by tagged *Streptomyces* will unravel part of the mechanisms involved in *Streptomyces*-mediated biocontrol. Moreover, the evidence of disease suppression by beneficial microbes *in vivo* encourages their development into bio-products for large-scale applications. However, the inconsistency between the biocontrol performance of BCAs in laboratory and in field occurred frequently and is considered one of the restraining factors of the biocontrol products (Velivellil et al., 2014). In addition, the application timing and method, as well as the concentration of BCAs play crucial roles in their biocontrol efficacy *in vivo* (Bonaterra et al., 2003; Fravel, 2005; Fernando et al., 2007; Müller and Berg, 2008).

In our previous study, two *Streptomyces* strains, *S. exfoliatus* FT05W and *S. cyaneus* ZEA17I, showed high *in vitro* inhibition of *S. sclerotiorum* (Bonaldi et al., 2015). The objective of this work was to evaluate their *in vivo* biological control activity against *S. sclerotiorum* on lettuce, assessing two different cell concentrations and two application timings in growth chamber, and subsequently their activity in field. Their performance in greenhouse and in field experiments was compared to *S. lydicus* WYEC 108, isolated from the commercial product Actinovate®. Simultaneously, we determined the colonization patterns of the EGFP-tagged *Streptomyces* on lettuce rhizoplane, using confocal laser scanning microscopy (CLSM) and we performed scanning electron microscopy (SEM) observations to verify the endophytic colonization of lettuce roots by EGFP- *S. exfoliatus* FT05W, the most promising strain. Finally, we determined the colonization dynamics by quantifying its concentration in lettuce rhizosphere and endorhiza at different times after lettuce inoculation.

## Materials and methods

### *Sclerotinia sclerotiorum* inoculum preparation

*Sclerotinia sclerotiorum* strain FW598 from the Plant Pathology Laboratory fungi repository, Department of Food, Environmental and Nutritional Sciences (DeFENS), University of Milan, was grown for 3 days at 20°C on Malt Extract Agar (MEA) medium (30 g/L Malt Extract, Difco, 15 g/L agar, Applichem). Then, ten agar-mycelium discs (6 mm diameter) were taken from the edge of an actively growing colony and transferred into a 300 mL flask containing 25 g of sterilized wheat kernels and 50 mL distilled water (Budge and Whipps, 2001). The flask was incubated for 3 weeks at 20°C and was regularly shaken. Afterwards, the pathogen-colonized wheat kernels were blended with 100 mL of sterilized water to obtain the “*S. sclerotiorum* slurry”. One gram of *S. sclerotiorum* slurry was diluted in an adequate volume of water to facilitate the distribution and added to 100 g of non-sterile Irish and Baltic peat-based growing substrate (Vigorplant, Piacenza, Italy). The inoculum density of *S. sclerotiorum* was estimated by plating serial dilutions on MEA medium. The plates were incubated at 20°C for 2 days, the number of colonies was counted and the inoculum density was calculated as CFU/g of slurry.

### *Streptomyces* biological control of lettuce drop in growth chamber experiment

Biological activity of the two *Streptomyces* strains, *S. exfoliatus* FT05W and *S. cyaneus* ZEA17I against *S. sclerotiorum* was first investigated *in vivo* in a growth chamber (24°C, 55% relative humidity and 15 h photoperiod) using plastic pots (Sterivent, Duchefa, Italy), 10 × 10 × 10 cm, filled with 200 g of inoculated growing substrate as mentioned above. *S. sclerotiorum* inoculum was ca. 3 × 10^4^ CFU/g of slurry. One mL of each *Streptomyces* strain spore suspensions (10^4^ CFU/mL or 10^6^ CFU/mL) was sprayed on the growing substrate immediately after the pathogen inoculation. Lettuce seeds, *Lactuca sativa* var. *capitata*, “Regina dei ghiacci”, (Semeurop, Italy) were sterilized in 2 mL of 0.7% sodium hypochlorite (NaOCl) for 5 min and were rinsed three times with sterilized water. Thirty seeds were sown in three rows in each pot at two different times. In the experiment A, lettuce was sown on the same day of substrate inoculation with *Streptomyces* strains and the pathogen. In the experiment B, lettuce was sown 7 days after the inoculation of *Streptomyces* strains and pathogen inoculation. *Streptomyces lydicus* WYEC 108, isolated from commercial product Actinovate® (Natural Industries, Inc. Houston), was used as the reference strain. The pot inoculated only with *S. sclerotiorum* was used as the inoculated control. The pot inoculated neither with *S. sclerotiorum* nor *Streptomyces* was used as the non-inoculated control. For experiments A and B, eight trials were prepared in three replicates: (1) non-inoculated control; (2) *S. sclerotiorum* inoculated control; (3) *S. exfoliatus* FT05W-10^4^ CFU/mL; (4) *S. exfoliatus* FT05W-10^6^ CFU/mL; (5) *S. cyaneus* ZEA17I-10^4^ CFU/mL; (6) *S. cyaneus* ZEA17I-10^6^ CFU/mL; (7) *S. lydicus* WYEC 108-10^4^ CFU/mL; (8) *S. lydicus* WYEC 108-10^6^ CFU/mL. Dead plants were counted from the emergence up to 18 days for the experiment A, and up to 25 days for the experiment B. Disease incidence was calculated as the percent of dead plants over the plants germinated in the non-inoculated control.

### *Streptomyces* biological control of lettuce drop in field experiment

Field experiment was carried out in Travacò Siccomario (Pavia, Italy), characterized by loamy soil. Lettuce, *Lactuca sativa* var. *capitata*, “Regina dei ghiacci” was grown in polystyrene seed trays (84 cells—48 cm^3^ each), filled with the non-sterile Irish and Baltic peat-based growing substrate described above. One seed was sown in each cell added with 0.5 mL of *Streptomyces* spore suspension (10^4^ CFU/mL) uniformly distributed on the growing substrate. Each tray was first covered with a thin layer of the growing substrate and then with coarse perlite. Two weeks later, the same amount of *Streptomyces* spore suspension was added to each cell. Three weeks after sowing, each cell was inoculated with 1 mL of *S. sclerotiorum* slurry (ca. 3 × 10^4^ CFU/g of slurry) prepared as described above. One day after the pathogen inoculation, the lettuce plants were transplanted into the field under plastic tunnel (width 1.2 m), at a density of 5.5 plants/m^2^. Five trials were prepared following a completely randomized block design in four replicates: (1) non-inoculated control; (2) *S. sclerotiorum* inoculated control; (3) *S. exfoliatus* FT05W; (4) *S. cyaneus* ZEA17I; (5) *S. lydicus* WYEC 108. Each trial consisted of 20 plants. Dead plants were counted at 3-week intervals, from the day the disease symptoms appeared until the end of the experiment. Disease incidence was calculated as the percent of dead plants over the number of transplanted plants.

### CLSM observations of lettuce root colonization by EGFP-*Streptomyces* strains

A Leica TCS SPE Confocal Laser Scanning Microscope (Leica Microsystems, Mannheim, Germany) equipped with solid state lasers for excitation was used to unravel lettuce root colonization patterns by the two *Streptomyces* strains, EGFP- *S. exfoliatus* FT05W and EGFP- *S. cyaneus* ZEA17I. Plant colonization assays were carried out at the Institute of Environmental Biotechnology, Graz University of Technology, Austria. The lettuce seeds were sterilized and bacterized with EGFP-tagged *Streptomyces* as previously described (Bonaldi et al., 2015). Subsequently, nine bacterized seeds were sown in three rows in a seed tray filled with 640 g of a mixture of autoclaved quartz sand (Scherf GmbH & Co. KG, Austria) and peat soil (“Gramoflor Profi-Substrat-Topfpikier M+Ton+Fe” GBC, Kalsdorf, Austria) in 1:3 ratio (w/w), and 200 mL of sterilized tap water were added. In two trays, no seeds were planted to monitor the soil moisture (≥25%) by a moisture analyzer (MB35 Halogen, Ohaus, USA). Nine surface sterilized non-bacterized seeds were sown in seed trays prepared in the same way and were used as non-inoculated control. After sowing, the seed trays were incubated in a growth chamber (24°C, 55% relative humidity and 14 h photoperiod). Two- and three-day-old seedlings and 2-week-old plants were used to verify the ability of EGFP-*Streptomyces* to colonize lettuce. At each interval, the roots of three bacterized plants, taken from a different seed tray, and one non-bacterized plant (negative control) were cleaned in sterile water and cut into 0.5 cm long sections for CLSM observation. Filter settings were adjusted to achieve the maximum signal from EGFP and low background autofluorescence of the plant tissues. The EGFP was excited with a 488 nm laser beam and the detection window was optimized for every field of view, in order to gain a better discrimination between the signal and the noise. Plant tissues were excited with a 635 nm laser beam and the autofluorescence emitted in the range 650–690 nm was recorded. The fluorescence signals from EGFP and from plant tissues were acquired sequentially. For each field of view, maximum projections of an appropriate number optical slices were acquired with a Z-step of 0.15–0.5 μm (“confocal stacks”) and the software Imaris 7.3 (Bitplane, Zurich, Switzerland) was used for post-processing (Erlacher et al., 2015).

### SEM observations of *Streptomyces* endophytic colonization

To further verify the endophytic *Streptomyces* colonization of lettuce roots, we carried out SEM observations (Leo Electron Microscopy, Cambridge, UK) at DeFENS, University of Milan, Italy, using the representative strain EGFP- *S. exfoliatus* FT05W, whose wild-type strain showed promising biocontrol potential. Inoculated and non-inoculated control lettuce seeds were grown in sterile conditions as described for CLSM observations with minor modification. Each seed was sown individually in a closed 200 mL box containing 80 g of a mixture of autoclaved sandy substrate (“Sabbia Vagliata” Gras Calce s.p.a., Italy) and peat soil (Vigorplant, Piacenza, Italy) in 1:3 ratio (w/w), and 20 mL sterilized tap water. Root samples from 1-, 2- and 3-week-old plants were harvested from two inoculated plants and one control plant. Root fragments, 1 cm long, were cut: in the proximity of soil surface, in the middle, and at the root apex. The fragments were rapidly frozen in liquid nitrogen, broken into pieces with the aid of two forceps (cryo-fractured) in order to expose the internal tissues, and prepared for SEM observations (Sardi et al., 1992; Rocchi et al., 2010). In total, 22 samples from 6 inoculated plants and 11 samples from three control plants were observed.

### Colonization dynamics in lettuce rhizosphere and endorhiza

To understand the competence of EGFP- *S. exfoliatus* FT05W to colonize lettuce rhizosphere and endorhiza, we exploited the introduced apramycin resistance marker to quantify the amount (as colony forming units, CFU) in sterile conditions as described by Bonaldi et al. (2015) for non-sterile conditions. Briefly: lettuce plants, obtained as described above for SEM observations, were collected at 1, 2, and 3 weeks after sowing. Seedlings with the whole root system were carefully extracted from the growth substrate and the bulk soil was removed by gently shaking the plants. Excised roots were immersed in 50 mL Falcon tubes containing 8–18 mL (volume varying according to plant age) of sterilized washing solution containing 0.9% NaCl (Sigma-Aldrich, United States) and 0.02% Silwet L-77 (Chemtura Manufacturing, Italy) and vortexed two-times for 15 s. The roots were removed and kept for inner root tissue analysis. The rhizosphere suspension was filtered through a 100 μm nylon mesh placed on the top of a Falcon tube, and centrifuged for 60 s to remove any remaining washing solution from the nylon mesh. The rhizosphere soil retained on the nylon mesh was collected and its dry weight was determined. The suspension was centrifuged at 10,600 g for 10 min and the pellet was resuspended in 2.5 mL of washing solution and plated in serial dilutions on Water Agar (WA) medium (15 g/L agar) added with 50 mg/L apramycin, 50 mg/L cycloheximide, and 50 mg/L nystatin. The plates were incubated at 24°C for 7 days. *Streptomyces* colonies were counted and the concentration was expressed as CFU/g of rhizosphere dry weight. For inner root tissues analysis, the roots were surface sterilized with propylene oxide for 1 h. Afterwards, they were washed in 2–3 mL of washing solution, depending on plant age, and 0.5 mL of the total volume of washing solution was plated on WA medium to verify the absence of contaminants. Subsequently, the roots were finely homogenized in the washing solution, left to macerate for 1 h and plated in serial dilutions on WA medium. The *Streptomyces* concentration was determined as described above and expressed as CFU/g of roots dry weight.

### Statistical analyses

All analyses were done using R software, version R3.0.2. (R_Core_Team, 2013). The data of the *in vivo* biological control experiments, concerning the activity of *Streptomyces* strains against *S. sclerotiorum*, were submitted to survival analysis by the survival package (Therneau, 2014). First, the time-to-death of lettuce plants untreated and treated with the streptomycetes was computed using the Kaplan-Meier method. Then, the estimated survivor curve of each *Streptomyces*-inoculated group was compared to the inoculated control via log-rank test (*P* = 0.05). Finally, the effect of each strain was quantified using the Cox proportional hazard model (Kleinbaum and Klein, 2012). This model computes the hazard *h* at time *t*, as follows: $h\left(t,X\right)={h_{0}\left(t\right)e}^{\sum_{i=1}^{p}\beta_{i}X_{i}}$ where *Xi* are the explanatory variables and ß*i* are the coefficients for each variable included in the model. The effect of each treatment was quantified as Hazard Ratio (*HR*) expressed as $\hat{HR}=exp\left[\overset{p}{\underset{i=1}{\sum }}\beta_{i\left(X_{i}^{*}{-X}_{i}\right)}\right]$ where X^*^ is the covariate for one group, generally the one with the larger hazard, and *X* for the group with the smaller hazard. The *HR* values equal to 1 were interpreted as no effect of *Streptomyces*-treated trial over *Streptomyces* non-treated control, *HR* > 1 means that the *Streptomyces* non-treated plants have a higher risk of lettuce drop and *HR* < 1 the opposite. The rhizosphere and endorhiza colonization dynamics data were submitted to ANOVA, followed by a Tukey *post hoc* test for multiple comparison (*P* = 0.05), using the TukeyC package (Faria et al., 2013).

## Results

### *Streptomyces* biological control of lettuce drop in growth chamber experiment

The germination rate of lettuce, calculated from the non-inoculated control, was 86.7%. When lettuce was sown the same day of the pathogen and *Streptomyces* inoculation (experiment A), the number of dead plants was recorded from the 4^th^ day after sowing to the 18^th^ day after sowing (Supplementary Table 1). The disease incidence of *S. sclerotiorum* inoculated control at the end of the experiment was 85% and none of the *Streptomyces* strains showed significant protection against lettuce drop according to both log-rank test and Cox model analysis (Table 1).

**Table 1:** **Biological control of ***Streptomyces*** strains against lettuce drop, when ***Lactuca sativa*** var. ***capitata***, “Regina dei ghiacci” was sown the same day of ***S. sclerotiorum*** and ***Streptomyces*** co-inoculation**.

**Trial**	**Disease Incidence (%)**	**Protection (%)**	**Log-rank test**	**Cox model**		
			***P*^b^**	**β^c^**	***HR*^d^**	***P*^e^**
*S. sclerotiorum* inoculated control	84.6	/^a^	/	/	/	/
*S. exfoliatus* FT05W (10^4^ CFU/mL)	92.3	−9.09	0.0952	−0.273	0.761 (0.544–1.065)	0.111
*S. exfoliatus* FT05W (10^6^ CFU/mL)	79.5	6.06	0.847	0.0171	1.017 (0.721–1.436)	0.923
*S. cyaneus* ZEA17I (10^4^ CFU/mL)	75.6	10.6	0.336	0.142	1.152 (0.812–1.635)	0.428
*S. cyaneus* ZEA17I (10^6^ CFU/mL)	83.3	1.52	0.864	0.0226	1.023 (0.727–1.439)	0.896
*S. lydicus* WYEC 108 (10^4^ CFU/mL)	76.9	9.09	0.399	−0.183	0.833 (0.594–1.168)	0.290
*S. lydicus* WYEC 108 (10^6^ CFU/mL)	88.5	−4.55	0.213	−0.233	0.792 (0.565–1.111)	0.177

a *No value*.

b *P-value of the log-rank test*.

c *β is the coefficient for the treatment covariate in the Cox model*.

d *Hazard Ratio (95% confidence interval)*.

e *P-value of the Cox model*.

When lettuce was sown 7 days after the pathogen and *Streptomyces* inoculation (experiment B), the dead plants were recorded from the 4^th^ day after sowing to the 25^th^ day after sowing (Supplementary Table 2). Disease incidence of the *S. sclerotiorum* inoculated control was 74.4% at the end of the experiment. The *Streptomyces* strains showed 25.7–51.7% protection of lettuce against *S. sclerotiorum*, which was statistically significant based on survival curves analyzed by log-rank test, except for *S. lydicus* WYEC 108 applied at the lower dose (10^4^ CFU/mL; *P* = 0.175). According to Cox regression model, *S. exfoliatus* FT05W, at both spore concentrations, significantly reduced the risk of lettuce drop disease, compared to the *S. sclerotiorum* inoculated control (*HR* = 2.078 and *HR* = 2.172, respectively). *S. cyaneus* ZEA17I was less effective than *S. exfoliatus* FT05W at both spore concentrations (*HR* = 1.595 and *HR* = 1.784, respectively). *S. lydicus* WYEC 108 applied at 10^6^ CFU/mL reduced the most the risk of lettuce drop (*HR* = 2.462), whereas when applied at 10^4^ CFU/mL, it was ineffective, which was in accordance with log-rank test analysis (*HR* = 1.261, *P* = 0.24, Table 2).

**Table 2:** **Biological control of ***Streptomyces*** strains against lettuce drop, when ***Lactuca sativa*** var. ***capitata***, “Regina dei ghiacci” was sown one week after ***S. sclerotiorum*** and ***Streptomyces*** co-inoculation**.

**Trial**	**Disease Incidence (%)**	**Protection (%)**	**Log-rank test**	**Cox model**		
			***P*^b^**	**β^c^**	***HR*^d^**	***P*^e^**
*S. sclerotiorum* inoculated control	74.4	/^*a*^	/	/	/	/
*S. exfoliatus* FT05W (10^4^ CFU/mL)	41.0	44.8	0.000942	0.731	2.078 (1.366–3.161)	0.000634
*S. exfoliatus* FT05W (10^6^ CFU/mL)	42.3	43.1	0.000337	0.776	2.172 (1.427–3.307)	0.000296
*S. cyaneus* ZEA17I (10^4^ CFU/mL)	53.9	27.6	0.0242	0.467	1.595 (1.072–2.372)	0.0212
*S. cyaneus* ZEA17I (10^6^ CFU/mL)	53.9	27.6	0.00523	0.579	1.784 (1.200–2.653)	0.00422
*S. lydicus* WYEC 108 (10^4^ CFU/mL)	55.1	25.7	0.175	0.232	1.261 (0.856–1.857)	0.24
*S. lydicus* WYEC 108 (10^6^ CFU/mL)	35.9	51.7	5.08E-05	0.901	2.462 (1.589–3.812)	5.43E-05

a *No value*.

b *P-value of the log-rank test*.

c *β is the coefficient for the treatment covariate in the Cox model*.

d *Hazard Ratio (95% confidence interval)*.

e *P-value of the Cox model*.

### *Streptomyces* biological control of lettuce drop in field experiment

Under field conditions, the number of dead plants was recorded from the 10^th^ to the 142^nd^ day after transplanting (Supplementary Table 3). At the end of the experiment, drop incidence of the *S. sclerotiorum* inoculated control was 50.0% and treatments with *S. exfoliatus* FT05W and *S. cyaneus* ZEA17I showed respectively 40.0% and 10.0% protection against lettuce drop (Supplementary Figure 1). Survival curves of lettuce treated with *S. exfoliatus* FT05W and *S. cyaneus* ZEA17I were not significantly different from the *S. sclerotiorum* inoculated control according to the log-rank test (Table 3). However, the *HR* used to estimate the effect of *S. exfoliatus* FT05W was 2.178, therefore the model estimated a risk of lettuce drop about two-times lower than that of the *S. sclerotiorum* inoculated control (*P* = 0.120). The survival curve of lettuce treated with *S. lydicus* WYEC 108 was significantly different from the *S. sclerotiorum* inoculated control (*P* = 0.0305), but with a negative protection of 30%. The HR of *S. lydicus* WYEC 108 was 0.448, confirming that plants inoculated only with *S. sclerotiorum* had significantly lower risk of drop compared to those treated with the *S. lydicus* WYEC 108 (*P* = 0.0309, Table 3).

**Table 3:** **Biological control of ***Streptomyces*** strains against lettuce drop of ***Lactuca sativa*** var. ***capitata***, “Regina dei ghiacci” under field conditions, Travacò Siccomario (Pavia, Italy)**.

**Trial**	**Disease incidence (%)**	**Protection (%)**	**Log-rank test**	**Cox model**		
			***P*^b^**	**β^c^**	***HR*^d^**	***P*^e^**
*S. sclerotiorum* inoculated control	50.0	/^a^	/	/	/	/
*S. exfoliatus* FT05W	30.0	40.0	0.802	0.779	2.178 (1.366–3.161)	0.120
*S. cyaneus* ZEA17I	45.0	10.0	0.939	0.0626	1.065 (1.427–3.307)	0.884
*S. lydicus* WYEC 108	65.0	−30.0	0.0305	−0.804	0.448 (1.072–2.372)	0.0309

a *No value*.

b *P-value of the log-rank test*.

c *β is the coefficient for the treatment covariate in the Cox model*.

d *Hazard Ratio (95% confidence interval)*.

e *P-value of the Cox model*.

### CLSM observations of lettuce root colonization by EGFP-*Streptomyces* strains

**Figure 1:**
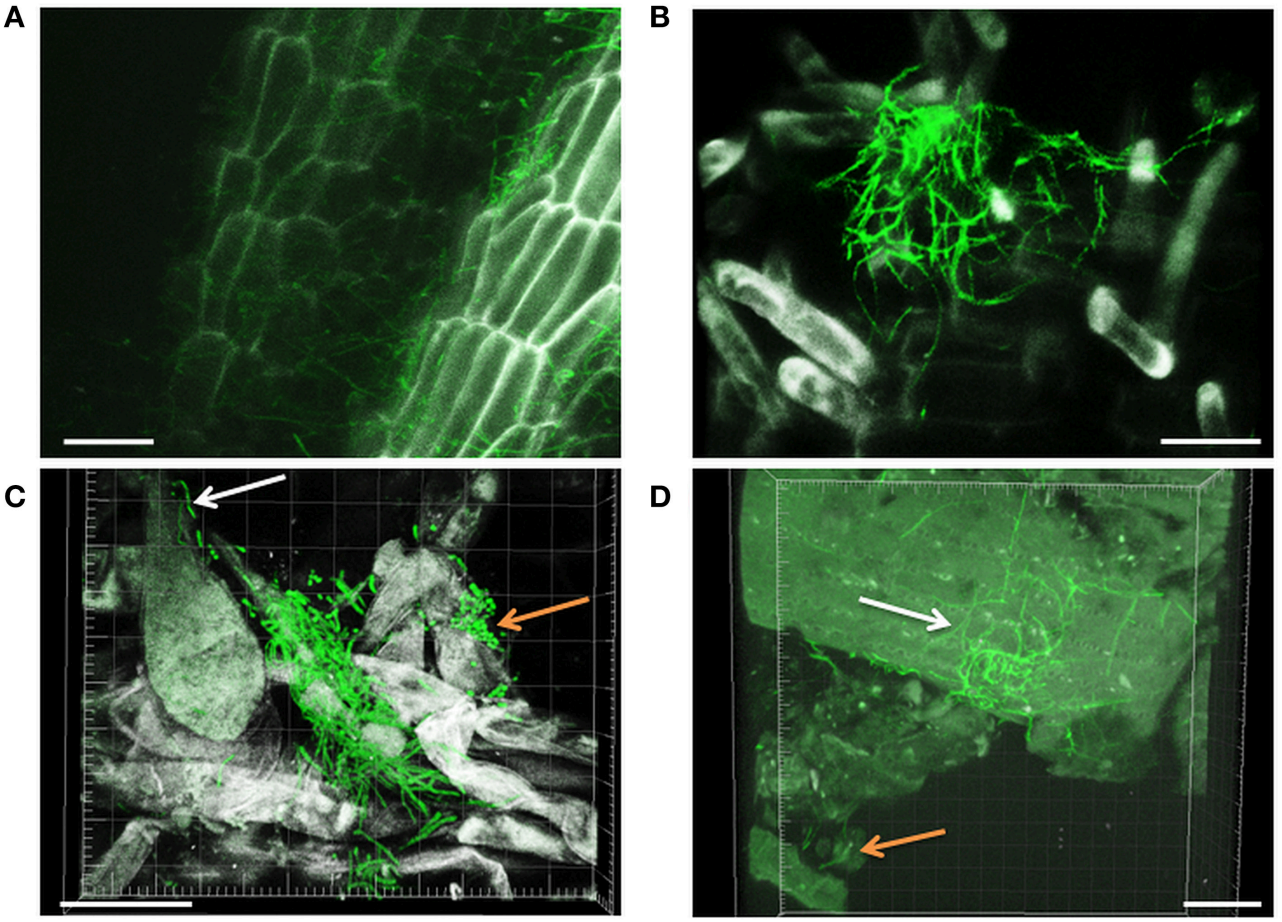
**CLSM observations of lettuce radicle colonization by EGFP-***Streptomyces***, two and three days after lettuce sowing**. Filamentous growth of EGFP- *S. cyaneus* ZEA17I at rhizoplane **(A)**, root hair zone **(B)**, and area close to the root hair zone **(C)**. The white arrow points to a single hypha, the orange arrow points to a group of germinating spores, **(D)** EGFP-*S. exfoliatus* FT05W colonizing lettuce root tissue with a soil particle attached (orange arrow). The white arrow points to the mycelium on the root surface, which is more abundant than that on the soil particle. Scale bar equals to 30 μm, for Figures **(A–D)**.

**Figure 2:**
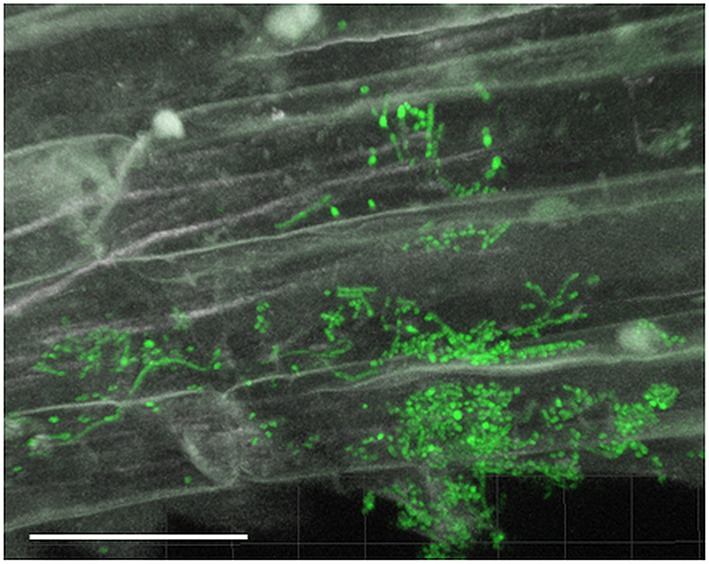
**CLSM observations of lettuce root colonization by EGFP-***Streptomyces*** two weeks after lettuce sowing**. Root surface colonization by EGFP-*S. cyaneus* ZEA17I. Scale bar equals to 30 μm.

Filamentous growth of EGFP-*Streptomyces* was frequently observed on the surface of 2- and 3-day-old lettuce roots (Figure 1) and the mycelium of EGFP-*S. cyaneus* ZEA17I colonized abundantly the lettuce rhizoplane (Figure 1A). The colonization by EGFP- *S. cyaneus* ZEA17I was observed mostly in the zone of cellular maturation of the main and lateral roots, and particularly on or in the proximity of root hairs (Figure 1B). Moreover, germinated spores grouped together in an area close to the root hair zone (Figure 1C). Interestingly, a piece of soil substrate that remained attached to the lettuce root tissue showed that EGFP- *S. exfoliatus* FT05W colonized more extensively the lettuce root surface than the soil particle (Figure 1D). We also observed EGFP-*Streptomyces* colonization on 2-week-old lettuce roots. In general, *Streptomyces* at different stages of their life cycle appeared concurrently at some sites of lettuce roots. Spores, single hyphae, spore chains, and mycelium of EGFP- *S. cyaneus* ZEA17I were observed on the root surface (Figure 2). We only rarely detected colonization on the root cap and elongation zone of the roots.

### SEM observations of lettuce root endophytic colonization by *Streptomyces* strains

**Figure 3:**
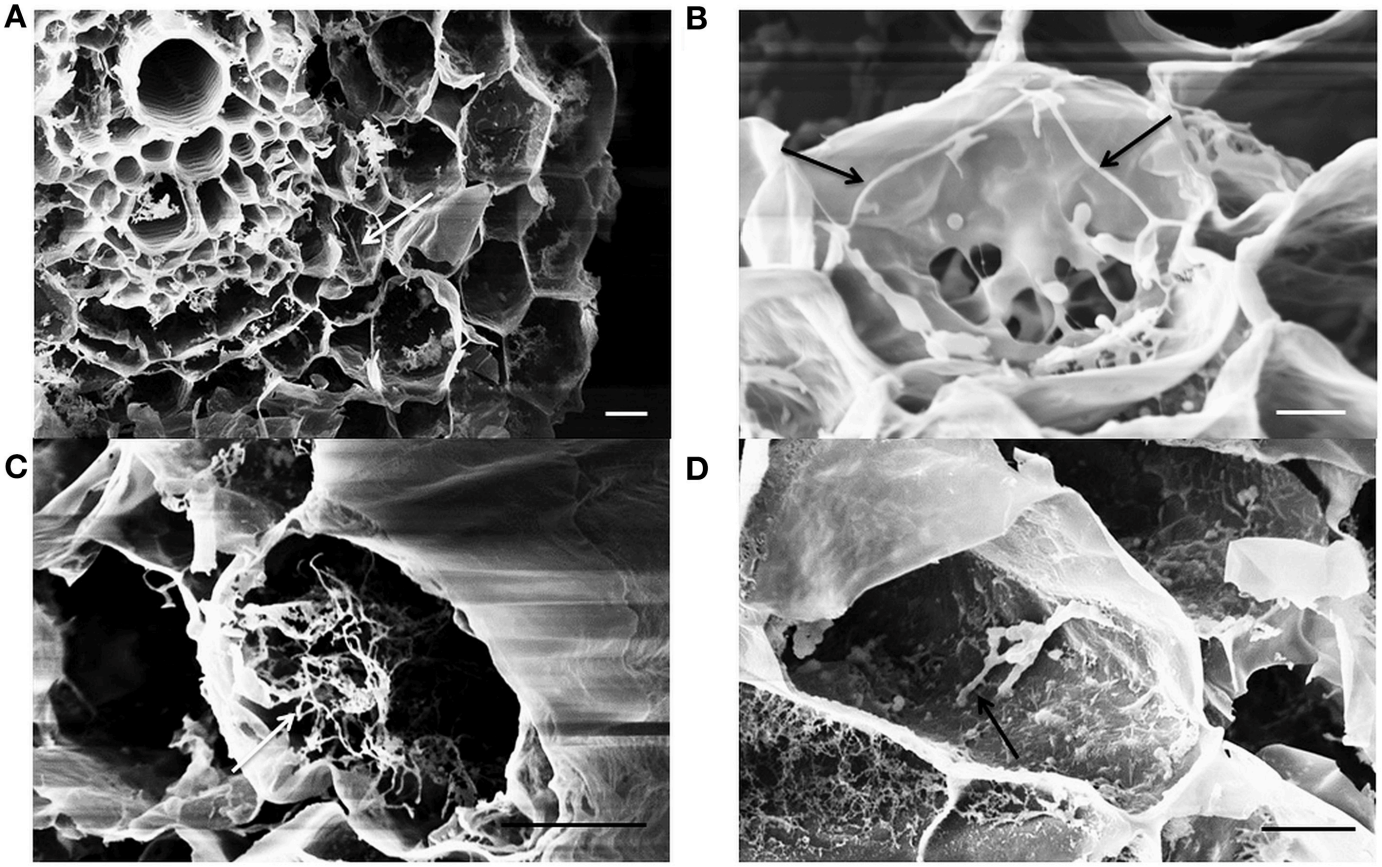
**SEM observations of lettuce root colonization by EGFP-***S. exfoliatus*** FT05W, one to three weeks after lettuce sowing**. Arrows indicate the hyphae inside one-week-old roots **(A)**, two-week-old roots **(B)**, 3-week-old roots **(C)**, a spore chain in the 3-week-old roots **(D)**. Scale bar equals to 10 μm **(A,C,D)** and 3 μm **(B)**.

Following sample cryo-fracturation, 88 sections were obtained and observed. Mycelium of EGFP- *S. exfoliatus* FT05W was frequently observed on the root surface of inoculated plants (micrograph not shown). Endophytic colonization of lettuce roots by EGFP- *S. exfoliatus* FT05W was observed in 99% of root sections from all samples from 1- to 3-week-old roots. Generally, several cells were colonized in each section (Figure 3). Along the entire length of the root, both close to the collar and near the apex, single hyphae were frequently detected inside cortical cells in 1-week-old (Figure 3A), and 2-week-old roots (Figure 3B), but not inside the vascular cylinder. In a few cases, mainly in 3-week-old roots, the hyphae grew abundantly inside cortical cells forming a tangled structure (Figure 3C). Hyphae growing inside cortical cells had a diameter of about 0.2 μm, half the size the ones grown on the root surface or *in vitro* cultures. EGFP- *S. exfoliatus* FT05W mainly colonized the endorhiza of lettuce as vegetative hyphae and rarely short spore chains were found (Figure 3D).

### *Streptomyces* colonization dynamics in lettuce rhizosphere and endorhiza

EGFP- *S. exfoliatus* FT05W showed stable concentration up to three weeks, both in lettuce rhizosphere and endorhiza, ranging from 1.72 × 10^6^ to 5.49 × 10^6^ CFU/g rhizosphere dry weight and from 1.10 × 10^5^ to 7.36 × 10^6^ CFU/g root dry weight, respectively (Table 4). There were no statistically significant differences in its concentration based on plant age both in rhizosphere and in endorhiza.

**Table 4:** **Colonization dynamics of EGFP-***S. exfoliatus*** FT05W in ***Lactuca sativa*** var. ***capitata***, “Regina dei ghiacci” rhizosphere and endorhiza**.

	**EGFP-*****S. exfoliatus*** **FT05W (CFU/g dry weight)**		
	**1 week**	**2 weeks**	**3 weeks**
Rhizosphere	1.72 × 10^6^ns^a^	2.45 × 10^6^ns	5.49 × 10^6^ns
Endorhiza	4.31 × 10^5^ns	7.36 × 10^6^ns	1.10 × 10^5^ns

a *ANOVA analysis, means in a row were not significantly different (P = 0.05)*.

## Discussion

Biological control strategies are gaining popularity in agriculture as a way to address some of the concerns about food security. Studies exploring novel biocontrol microorganisms and investigating their mechanisms of action have consistently increased. However, we are still facing significant fluctuations in the efficiency of biocontrol microorganisms, which represent a critical limitation to a more general and broader use in agriculture as plant protection products. The variable performance of BCAs could be due to the limited knowledge about their mode of action and about their ability to survive and establish stable relation with plant host (Compant et al., 2005; Cuppels et al., 2013). Even when they successfully establish symbiosis with the host, e.g., in the rhizosphere, another challenge is whether the beneficial microbes can compete and suppress the pathogens. Nowadays, tremendous efforts are increasingly done to investigate the colonization patterns of BCAs on plants and their biocontrol against pathogens *in vivo* (Chen et al., 2013; Xue et al., 2013; Maldonado-Gonzalez et al., 2015a,b; Santiago et al., 2015).

In this work we have studied *in vivo* biocontrol activity of two promising *Streptomyces* strains against *S. sclerotiorum* as a follow up of a previous study, in which we obtained excellent *in vitro* activity against this pathogen (Bonaldi et al., 2015). *S. lydicus* WYEC 108, reisolated from the commercial product Actinovate®, recommended for the management of several soil borne fungal pathogens including *S. sclerotiorum* (Yuan and Crawford, 1995; Leisso et al., 2009; Zeng et al., 2012), was incorporated to our growth chamber and field experiments as the reference strain. In growth chamber experiments, we tested two different timings of *Streptomyces* application and two different spore concentrations. Both microorganisms, the pathogen and the *Streptomyces* strains, were inoculated at the same time of lettuce sowing, or 7 days before the lettuce sowing. The obtained results clearly showed that the timing of *Streptomyces* application has a significant impact on their biocontrol activity. In particular, both *S. exfoliatus* FT05W and *S. cyaneus* ZEA17I significantly reduced *S. sclerotiorum* lettuce drop when they were applied 7 days before the plant sowing. In contrast, when application of *S. sclerotiorum* and *Streptomyces* was postponed until sowing, neither strain was able to reduce disease incidence and improve lettuce survival. The importance of application timing on biocontrol activity of BCAs against apple diseases was also reported for blue mold, bitter rot, and apple scab, as well as for brown rot of cherries and plums (Teixido et al., 1999; Poleatewich et al., 2012; Rungjindamai et al., 2014). For *Streptomyces*, the production of biocontrol related secondary metabolites is induced or increased when the aerial hyphae appear and sporulation starts (Hopwood, 1988; Chater, 1996; Pope et al., 1996). Therefore, we hypothesize that *Streptomyces* need time after application to perform biocontrol activity. We also observed that the efficacy of some strains was positively correlated to the application rate, as for the reference strain *S. lydicus* WYEC 108, whose protection reached 51.7% when applied at 10^6^ CFU/mL. In the field experiment, the three *Streptomyces* strains showed different biological control activity against *S. sclerotiorum* and the results were not always consistent with those observed in the greenhouse. *S. exfoliatus* FT05W was able to reduce drop incidence by 40%, which can be considered a promising biocontrol performance compared to other studies. For instance, application of *S. padanus* SS-07 resulted in 17% reduction of *Rhizoctonia* damping-off on Chinese cabbage, while four *Streptomyces* spp. strains showed 2 to 9.8% reduction of *Verticillium* wilt on eggplant (Chung et al., 2005; Bubici et al., 2013). On the contrary, *S. lydicus* WYEC 108 had a surprisingly negative effect on lettuce drop incidence in the field, opposite to the 51.7% protection obtained in the growth chamber experiment. One possible explanation could be that in the rhizosphere, the microbes respond to the many metabolites released by plant roots, as well as to the natural microflora producing a variety of compounds (Morgan et al., 2005). Such complex interactions, especially under field conditions, may result in positive, neutral, and negative effects on plant growth, health, and survival (Bouwmeester et al., 2007; Berg, 2009). Negative effect of *S. lydicus* WYEC 108 was previously reported for tomato bacterial spot as well as for tomato early blight in Canada (Cuppels et al., 2013). However, in the same study, a combined application of *S. lydicus* WYEC108 and *P. fluorescens* A506 resulted in a good protection against the two diseases. Similarly, *S. lydicus* WYEC 108 applied to control *Fusarium* wilt of watermelon resulted in increased disease severity in American soils, whereas the combination of green manure and *S. lydicus* WYEC 108 mitigated the negative effect. *S. lydicus* inefficacy was probably due to its lack of survival on watermelon roots in those specific conditions (Himmelstein et al., 2014). Another hypothesis might be that under certain environmental conditions *S. lydicus* WYEC 108 produces fungal growth promoting secondary metabolites, which enhance the pathogen growth and promote the infection of the host plant. Fungal growth promotion was shown for *Streptomyces* sp. strain AcH 505 producing auxofuran, a molecule which improved mycelial growth of the ectomycorrhizal fungus, *Amanita muscaria*, and its interaction with spruce (Schrey et al., 2005; Riedlinger et al., 2006).

The beneficial plant-microbe interactions occurring at specific sites usually require the microbe competence for host colonization (Berg et al., 2015; Hardoim et al., 2015). It has been hypothesized that the *Streptomyces*-mediated disease suppression is linked to the production of active secondary metabolites and their ability to colonize plant roots (Tokala et al., 2002; Franco et al., 2007). In this study, we investigated *Streptomyces* lettuce colonization as one of the characters underlying *Streptomyces*-mediated biocontrol. The use of fluorescent proteins to study plant colonization by BCAs such as *Bacillus* and *Pseudomonas* spp. has been widely reported (Buddrus-Schiemann et al., 2010; De-Bashan et al., 2010; Krzyzanowska et al., 2012; Sun et al., 2014). However, very few studies investigated *Streptomyces* colonization patterns on plants using fluorescent proteins in combination with CLSM. Coombs and Franco (2003) demonstrated that the EGFP-tagged endophytic *Streptomyces* sp. strain EN27 rapidly colonized the wheat embryo, as it was detected in developing seeds as early as 24 h after inoculation, but long-term rhizosphere competence and root colonization were not investigated. Similarly, Joshi et al. (2007) labeled a pathogenic strain of *S. turgidiscabies* with EGFP, and it was detected mainly on the surface of several-day-old radish seedlings, without any further monitoring. In our study, both EGFP-*S. exfoliatus* FT05W and EGFP-*S. cyaneus* ZEA17I were able to rapidly colonize the lettuce root system, and establish interactions with the host from early stages of seed germination and root development. Although it is not known if the localization of *Streptomyces* regulates their activity for biological control of pathogens, it has been hypothesized that endophytic bacteria form more stable interactions with plants than rhizospheric or epiphytic bacteria (Ryan et al., 2008; Compant et al., 2010; Malfanova et al., 2011). Using CLSM, we were able to detect EGFP-*Streptomyces* extensively colonizing the rhizoplane, and the SEM analyses confirmed the presence of EGFP-*S. exfoliatus* FT05W on the root surface and revealed the endophytic colonization in the root cortex. To our knowledge, this is the first study, which describes the observation of lettuce epiphytic and endophytic colonization by EGFP-tagged *Streptomyces* up to three weeks. In addition, we consistently recovered high concentration of EGFP-*S. exfoliatus* FT05W (10^5^-10^6^ CFU/g dry weight) from both, lettuce rhizosphere and endorhiza, up to three weeks after seed inoculation. This evidence allows us to conclude that *S. exfoliatus* FT05W is both rhizospheric and endophytic in lettuce roots.

The ability of microorganisms to colonize plant roots enables them to establish long-term beneficial interactions including biocontrol against plant pathogens (Adesina et al., 2009; Schreiter et al., 2014). The ability of *S. exfoliatus* FT05W to produce chitinases, to solubilize phosphates and to synthesize IAA (Bonaldi et al., 2015) coupled with its stable rhizosphere competence and endophytic colonization of lettuce roots determined in this study, could explain its biocontrol activity against *S. sclerotiorum*. When *S. exfoliatus* FT05W was applied 1 week before plant sowing, it showed significant protection against lettuce drop in growth chamber, data that have been confirmed in field. Studying the colonization patterns of *Streptomyces* on lettuce in the presence of the pathogen will give us insight into whether and how *Streptomyces* spp. compete with plant pathogens, leading to better understanding of *Streptomyces*-mediated biocontrol. In addition, studies evaluating *S. exfoliatus* FT05W activity against other soil borne fungal pathogens (e.g., *Fusarium, Pythium, Rhizoctonia*, or *Verticillium* spp.) and its ability to establish stable interactions with other hosts are needed to make it more attractive for its development into a commercial biocontrol product.

## Author contributions

XC performed the CLSM observations, promoted the SEM and colonization dynamics studies, and drafted the manuscript. CP evaluated the colonization dynamics data and conducted the statistical analyses. MB performed the biocontrol experiments in greenhouse and in field. MS performed SEM sample preparation, observations and acquired SEM pictures. AE contributed to the CLSM observations and post-processed the CLSM photos. AK participated to the discussions of each section of experiments, and improved the manuscript. GB hosted and supported XC in her lab to perform the CLSM observations with assistance of AE. PC designed the outline of the study and partly supported the research. All authors read and approved the final manuscript.

### Conflict of interest statement

The authors declare that the research was conducted in the absence of any commercial or financial relationships that could be construed as a potential conflict of interest.

## Supplementary material

Supplementary Figure 1 **Survival of lettuce plants (*Lactuca sativa* var. *capitata*, “Regina dei ghiacci”) in the field experiment (ca. 60 days after transplanting) inoculated with (A) *Sclerotinia sclerotiorum* (inoculated control); (B) *S. sclerotiorum* + *S. exfoliatus* FT05W; (C) *S. sclerotiorum* + *S. cyaneus* ZEA17I; and (D) *S. sclerotiorum* + *S. lydicus* WYEC 108**.

Supplementary Table S1 **Number of lettuce dead plants recorded for the experiment A, when *Lactuca sativa* var. *capitata*, “Regina dei ghiacci” was sown the same day of *S. sclerotiorum* and *Streptomyces* co-inoculation**.

Supplementary Table S2 **Number of lettuce dead plants recorded for the experiment B, when *Lactuca sativa* var. *capitata*, “Regina dei ghiacci” was sown one week after *S. sclerotiorum* and *Streptomyces* co-inoculation**.

Supplementary Table S3 **Number of *Lactuca sativa* var. *capitata*, “Regina dei ghiacci” dead plants recorded for field experiment, Travacò Siccomario (Pavia, Italy)**.
