# Supplementary figures and images for: Biological Control of Lettuce Drop and Host Plant Colonization by Rhizospheric and Endophytic Streptomycetes
Source: Front Microbiol. 2016 May 20;7:714. doi: 10.3389/fmicb.2016.00714 (PMC4874062; doi:10.3389/fmicb.2016.00714)

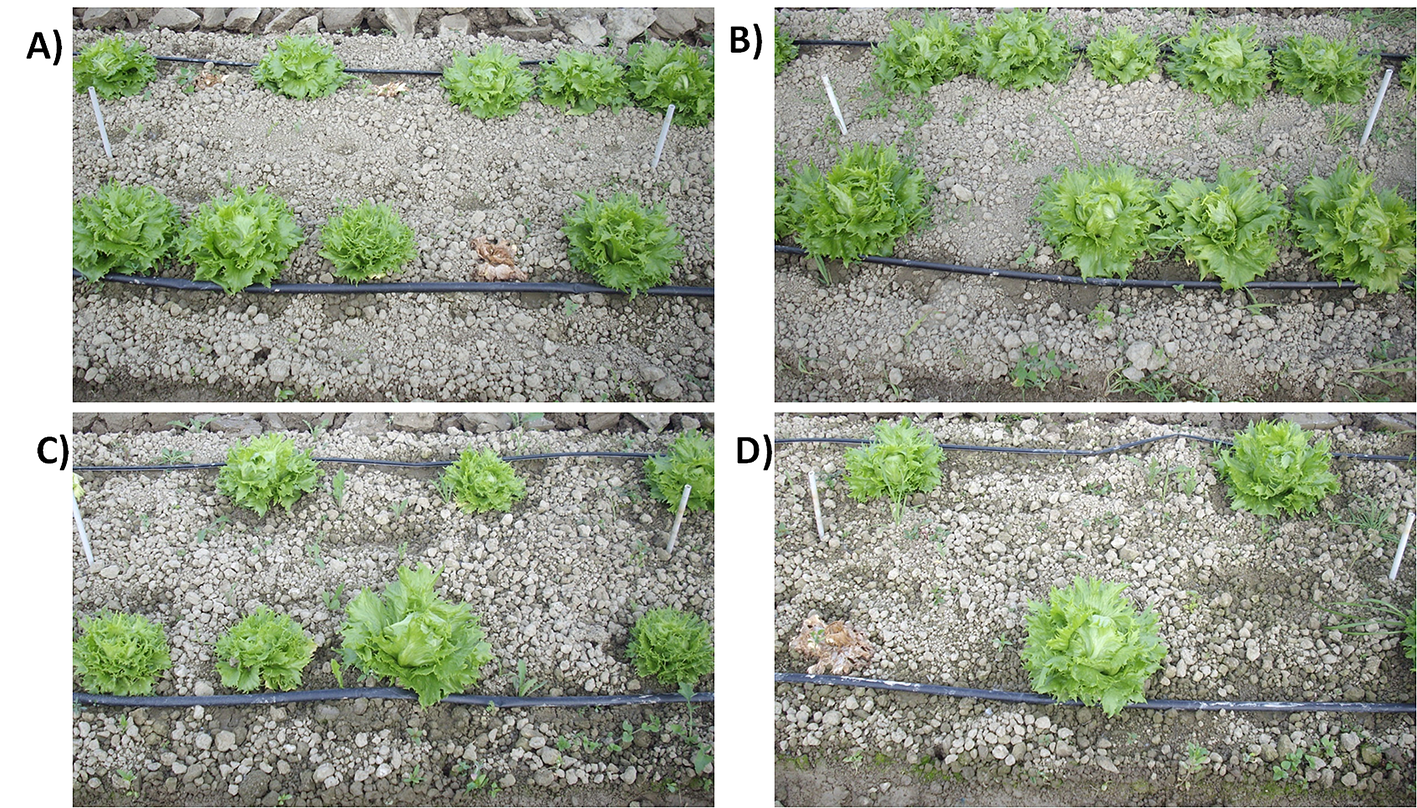

Supplement: Supplementary Figure 1 — Survival of lettuce plants (Lactuca sativa var. capitata, “Regina dei ghiacci”) in the field experiment (ca. 60 days after transplanting) inoculated with (A) Sclerotinia sclerotiorum (inoculated control); (B) S. sclerotiorum + S. exfoliatus FT05W; (C) S. sclerotiorum + S. cyaneus ZEA17I; and (D) S. sclerotiorum + S. lydicus WYEC 108. [file Image1.TIF]
