# Supplementary material for: Biological Control of Lettuce Drop and Host Plant Colonization by Rhizospheric and Endophytic Streptomycetes
Source: Front Microbiol. 2016 May 20;7:714. doi: 10.3389/fmicb.2016.00714 (PMC4874062; doi:10.3389/fmicb.2016.00714)
Supplement: Supplementary Table S1 — Number of lettuce dead plants recorded for the experiment A, when Lactuca sativa var. capitata, “Regina dei ghiacci” was sown the same day of S. sclerotiorum and Streptomyces co-inoculation. [file Table1.DOCX]

Supplementary table 1: Number of lettuce dead plants recorded for the experiment A, when *Lactuca sativa* var. *capitata*, “Regina dei ghiacci” was sown the same day of *S. sclerotiorum* and *Streptomyces* co-inoculation.

|  | Dai^1^ | | | | | | | | |
| --- | --- | --- | --- | --- | --- | --- | --- | --- | --- |
| Trial | 4 | 7 | 8 | 9 | 10 | 11 | 14 | 16 | 18 |
| *S. sclerotiorum* inoculated control | 21 | 26 | 2 | 4 | 0 | 3 | 5 | 4 | 1 |
| *S. exfoliatus* FT05W (10^4^ CFU/mL) | 20 | 29 | 7 | 10 | 0 | 2 | 4 | 0 | 0 |
| *S. exfoliatus* FT05W (10^6^ CFU/mL) | 19 | 30 | 7 | 2 | 3 | 0 | 1 | 0 | 0 |
| *S. cyaneus* ZEA17I (10^4^ CFU/mL) | 18 | 30 | 5 | 2 | 1 | 3 | 0 | 0 | 0 |
| *S. cyaneus* ZEA17I (10^6^ CFU/mL) | 15 | 27 | 16 | 3 | 1 | 1 | 1 | 0 | 1 |
| *S. lydicus* WYEC 108 (10^4^ CFU/mL) | 14 | 30 | 11 | 2 | 0 | 0 | 1 | 1 | 1 |
| *S. lydicus* WYEC 108 (10^6^ CFU/mL) | 24 | 33 | 5 | 3 | 0 | 2 | 1 | 0 | 1 |

^1^Days after inoculation.
