# Supplementary material for: Biological Control of Lettuce Drop and Host Plant Colonization by Rhizospheric and Endophytic Streptomycetes
Source: Front Microbiol. 2016 May 20;7:714. doi: 10.3389/fmicb.2016.00714 (PMC4874062; doi:10.3389/fmicb.2016.00714)
Supplement: Supplementary Table S2 — Number of lettuce dead plants recorded for the experiment B, when Lactuca sativa var. capitata, “Regina dei ghiacci” was sown one week after S. sclerotiorum and Streptomyces co-inoculation. [file Table2.DOCX]

Supplementary table 2: Number of lettuce dead plants recorded for the experiment B, when *Lactuca sativa* var. *capitata*, “Regina dei ghiacci” was sown one week after *S. sclerotiorum* and *Streptomyces* co-inoculation.

| Trial | Dai^1^ | | | | | | | | | |
| --- | --- | --- | --- | --- | --- | --- | --- | --- | --- | --- |
|  | 4 | 7 | 8 | 9 | 10 | 11 | 14 | 16 | 18 | 25 |
| *S. sclerotiorum* inoculated control | 20 | ^8^ | 0 | 0 | 2 | 1 | 5 | 2 | 0 | 20 |
| *S. exfoliatus* FT05W (10^4^ CFU/mL) | 11 | 3 | 2 | 1 | 0 | 2 | 6 | 5 | 0 | 2 |
| *S. exfoliatus* FT05W (10^6^ CFU/mL) | 12 | 0 | 4 | 0 | 2 | 2 | 3 | 1 | 0 | 9 |
| *S. cyaneus* ZEA17I (10^4^ CFU/mL) | 19 | 6 | 3 | 0 | 0 | 0 | 2 | 3 | 0 | 9 |
| *S. cyaneus* ZEA17I (10^6^ CFU/mL) | 10 | 4 | 1 | 1 | 3 | 0 | 5 | 5 | 0 | 13 |
| *S. lydicus* WYEC 108 (10^4^ CFU/mL) | 33 | 2 | 0 | 0 | 2 | 0 | 0 | 0 | 0 | 6 |
| *S. lydicus* WYEC 108 (10^6^ CFU/mL) | 13 | 0 | 2 | 0 | 2 | 3 | 0 | 2 | 0 | 6 |

^1^Days after inoculation.
