# Supplementary material for: Biological Control of Lettuce Drop and Host Plant Colonization by Rhizospheric and Endophytic Streptomycetes
Source: Front Microbiol. 2016 May 20;7:714. doi: 10.3389/fmicb.2016.00714 (PMC4874062; doi:10.3389/fmicb.2016.00714)
Supplement: Supplementary Table S3 — Number of Lactuca sativa var. capitata, “Regina dei ghiacci” dead plants recorded for field experiment, Travacò Siccomario (Pavia, Italy). [file Table3.DOCX]

Supplementary table 3: Number of *Lactuca sativa* var. *capitata*, “Regina dei ghiacci” dead plants recorded for field experiment, Travacò Siccomario (Pavia, Italy).

| Trial | Days^1^ | | | | | | | |
| --- | --- | --- | --- | --- | --- | --- | --- | --- |
|  | 10 | 16 | 20 | 30 | 45 | 73 | 120 | 142 |
| *S. sclerotiorum* inoculated control | 3 | 1 | 1 | 0 | 2 | 0 | 3 | 0 |
| *S. exfoliatus* FT05W | 3 | 2 | 0 | 0 | 0 | 1 | 0 | 0 |
| *S. cyaneus* ZEA17I | 7 | 2 | 0 | 0 | 0 | 0 | 0 | 0 |
| *S. lydicus* WYEC | 7 | 1 | 0 | 2 | 2 | 0 | 1 | 0 |

^1^ Days after transplanting.
